# NAVIGATING REAL-WORLD BARRIERS IN NURSING HOME PRAGMATIC TRIALS

**DOI:** 10.1093/geroni/igae098.1370

**Published:** 2024-12-31

**Authors:** A Lynn Snow, A Lynn Snow

**Affiliations:** Chair; Discussant

## Abstract

Nursing homes (NHs) are an important setting for the use of pragmatic clinical trial (PCT) approaches, particularly due to the challenging implementation environments since the COVID-19 pandemic. NHs have well documented, long-standing quality challenges, staffing shortages, and substantial time gaps between the development of new evidence and innovations and their wide-spread industry adoption. Thus, NHs are an opportune setting for the development of pragmatic methods to accelerate the adoption of efficacious interventions into practice. In this symposium we explore successful approaches used in current NH PCTs to address five common challenges: organizational partnerships, intervention adaptation, use of technology in PCTs, pragmatic measurement of outcomes, and evaluating intervention-related staff competencies. We will explore each topic through an example from one or two recent NH PCTs, sharing challenges and successes. The discussant and panel will then examine the topics across all four represented PCTs and consider applications to future PCTs.

